# Application of digital health technologies in hypertension self-management: a narrative review

**DOI:** 10.3389/fpubh.2026.1865773

**Published:** 2026-06-04

**Authors:** Sicheng Zhao, Yue Yang, Ning Lü

**Affiliations:** 1 First Clinical Medical College, Heilongjiang University of Chinese Medicine, Harbin, China; 2 First Affiliated Hospital, Heilongjiang University of Chinese Medicine, Harbin, China

**Keywords:** artificial intelligence, digital health, hypertension, lifestyle intervention, medication adherence, mobile health, self-management, wearable devices

## Abstract

Hypertension remains a major global public health challenge and a leading risk factor for cardiovascular morbidity and mortality. Effective long-term control of BP largely depends on sustained patient engagement, medication adherence, and lifestyle modification. However, traditional care models often face limitations in delivering continuous monitoring, personalized support, and long-term behavioral interventions. This review provides a comprehensive overview of digital health interventions—including mobile health (mHealth) applications, wearable devices, and artificial intelligence (AI)-driven tools—in the management of hypertension. Current evidence from randomized controlled trials and observational studies suggests that these technologies can significantly improve medication adherence, enhance self-monitoring of BP, and promote healthier behaviors such as increased physical activity and dietary modification. In particular, mHealth applications incorporating reminders, feedback, and educational components have demonstrated measurable improvements in adherence and BP control. Wearable devices enable real-time physiological monitoring, while AI-based systems offer opportunities for personalized risk prediction and adaptive intervention strategies. Despite these promising findings, several challenges remain. The long-term effectiveness and sustainability of digital health interventions are still not well established, with many studies limited to short follow-up periods. In addition, issues related to interoperability between different digital platforms, data privacy and security concerns, and unequal access to technology may hinder widespread implementation. Variability in study design and intervention components also limits the comparability of findings across studies. Future research should focus on conducting large-scale, long-term trials to evaluate clinical outcomes and cost-effectiveness. Efforts are also needed to improve system integration, enhance user engagement, and ensure equitable access to digital health technologies. Overall, digital health interventions represent a promising and scalable approach to improving hypertension self-management and supporting more efficient, patient-centered care.

## 1 Introduction

This review aims to provide an overview of the current landscape of hypertension self-management, summarize the major digital health tools and technological frameworks currently in use, and examine the key challenges associated with their implementation.

**Figure 1:**
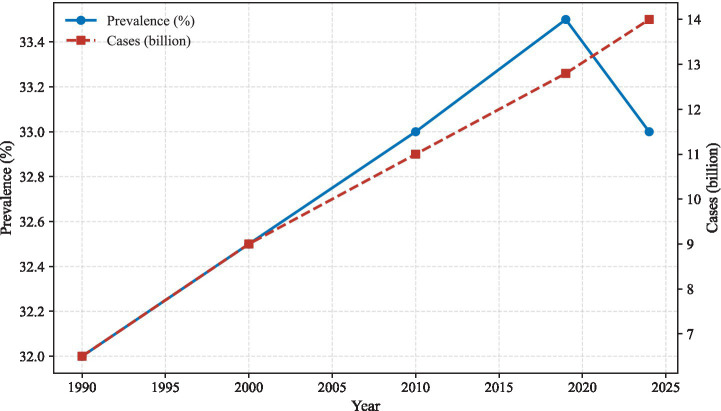
Global trends in hypertension prevalence, 1990–2024. Data were obtained from the Global Burden of Disease Study 2021 (GBD 2021). Age-standardized rates were calculated using the GBD standard population.

**Figure 2:**
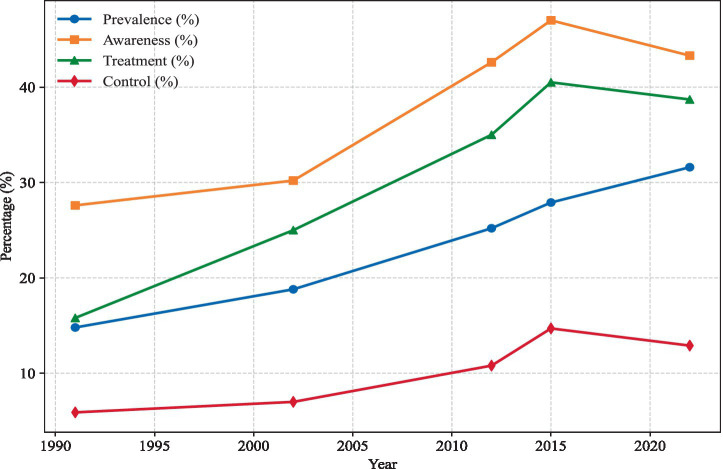
Trends in hypertension prevalence in China, 1991–2022. Trends in hypertension prevalence, awareness, treatment, and control in China from 1991 to 2022. Data were derived from national epidemiological surveys and large-scale population studies, including the China Hypertension Survey and China CDC reports. Intermediate values were estimated using interpolation.

Hypertension remains a major global public health challenge. According to a World Health Organization report (2025) (1), the number of individuals living with hypertension has remained at approximately 1.3–1.4 billion over the past 5 years, with a gradual upward trend,affecting nearly one-third of adults aged 30–79 years. As shown in Figure 1, global trends in hypertension prevalence from 1990 to 2024 demonstrate overall variation over time, with differing trajectories observed across study periods. The condition contributes substantially to mortality and disability worldwide and continues to impose a significant burden on healthcare systems (2, 3). In China, hypertension prevalence remains high. The Report on Cardiovascular Health and Diseases in China (2023) indicates that the prevalence of hypertension among adults aged ≥18 years is approximately 27.9%, corresponding to about 245 million individuals (4, 5). As shown in Figure 2, trends in hypertension prevalence in China from 1991 to 2022 demonstrate an overall upward pattern over time. In addition, the urban–rural gap shows temporal variation, with a more rapid increase observed in rural populations in recent years (6, 7).

Effective self-management is considered a key strategy for delaying disease progression and improving clinical outcomes. Traditional management approaches, primarily based on health education and periodic follow-up, have shown limited effectiveness (8), as awareness, treatment, and control rates remain suboptimal. These approaches are often constrained by poor adherence and clinical inertia, making it difficult to achieve sustained and individualized management (9).

In recent years, digital health technologies have been increasingly applied in chronic disease management. By leveraging wearable devices, mobile health applications, and artificial intelligence, these approaches enable real-time monitoring, personalized health education, and clinical decision support. In addition, feedback-driven behavioral interventions may enhance patient engagement. Such technologies offer advantages in terms of precision, adaptability, and scalability, allowing for more tailored interventions across diverse patient populations (10, 11). A recent systematic review and meta-analysis conducted in low- and middle-income countries reported that digital health interventions were associated with improvements in BP control, medication adherence, and lifestyle behaviours among patients with hypertension. However, variations in study design, intervention duration, and sample size across the included studies suggest that the overall strength and long-term generalizability of the evidence should be interpreted cautiously (12).

## 2 Methods

As this study was designed as a narrative review, a formal systematic review protocol such as PRISMA was not applied. Nevertheless, a structured approach was adopted to ensure a comprehensive and balanced overview of the relevant literature.

Electronic searches were conducted in PubMed, Scopus, and Web of Science databases for studies published between January 2010 and May 2025. The search strategy combined free-text keywords and controlled vocabulary terms related to hypertension and digital health interventions. Search terms were developed with reference to Medical Subject Headings (MeSH) to improve the comprehensiveness and consistency of the retrieval process. The search strategy included terms related to “hypertension,” “high blood pressure,” “digital health,” “mobile health,” “mHealth,” “wearable devices,” “artificial intelligence,” “telemedicine,” and “self-management.” Boolean operators (“AND” and “OR”) were applied to refine the search strategy. The reference lists of relevant reviews and key articles were manually screened to identify additional studies.

All records retrieved from the database search were imported into a reference management system, and duplicate records were removed prior to screening. Study selection was performed through a two-stage process involving title and abstract screening followed by full-text review. To reduce selection bias, screening was independently conducted by two reviewers, with disagreements resolved through discussion or consultation with a third reviewer.

Eligible publications included peer-reviewed original research articles, systematic reviews, and meta-analyses published in English. In addition, reports from authoritative public health organizations, including the World Health Organization, were consulted to provide contextual epidemiological data and policy insights.

Studies were included if they focused on the application of digital health technologies in hypertension management, particularly in relation to self-management behaviors, medication adherence, blood pressure monitoring, or lifestyle modification. Studies were excluded if they lacked relevance to digital health interventions for hypertension management, were duplicate publications, conference abstracts without full-text availability, or non-English language articles.

Approximately 20 studies were reviewed in full for narrative synthesis. Given the narrative nature of this review, the aim was not to provide an exhaustive or quantitative synthesis of all available evidence, but rather to identify representative studies reflecting key developments, application pathways, and implementation challenges in this field. Representative studies were selected based on methodological quality (e.g., randomized controlled trials, systematic reviews, and meta-analyses), sample size, recency, and relevance to clinical practice. Therefore, a PRISMA-style flow diagram and formal risk-of-bias assessment were not conducted.

## 3 Hypertension management patterns across different age-stratified populations

Hypertension management is a multifaceted process involving pharmacological treatment, dietary regulation, physical activity, and health education. However, substantial heterogeneity exists in self-management capacity across different age groups. Despite advances in treatment strategies, overall BP control and self-management remain suboptimal.

Evidence from a large epidemiological study by Lewington et al. (13)demonstrated a continuous, positive association between BP levels and cardiovascular mortality in China, with risk increasing progressively alongside both elevated BP and advancing age, and no clear threshold observed. These findings suggest that the risk of hypertension-related cardiovascular events rises with age, underscoring the importance of maintaining effective BP control across different age groups. A study conducted in South Korea (14) found that younger individuals tend to have lower BP control rates and weaker disease awareness compared with older adults. Older patients are more likely to engage in regular BP monitoring and community-based health services, whereas younger populations are more frequently exposed to unhealthy dietary patterns and sedentary lifestyles. These behavioral differences may contribute to disparities in hypertension control across age groups. In addition, although cardiovascular risk increases with age, this dynamic change is not yet fully reflected in current risk stratification systems (15). Similar patterns have been observed in China. A large population-based study (16) reported a hypertension control rate of 23.36% among individuals aged 45 years and older. Control rates increased with age and peaked in the 85–94 age group, but declined again among those aged 95 years and older. Collectively, these findings suggest a potentially complex and non-linear relationship between age and BP control, a pattern that has not been systematically synthesized in previous literature. This observation may reflect the combined influence of multiple age-related factors, including disease awareness, lifestyle behaviors, physical functioning, digital literacy, and self-management capacity, which may differentially affect BP management across age groups. With advancing age, declines in physical function, cognitive ability, and health literacy may compromise patients ability to adhere to complex treatment regimens, highlighting the need for additional support through digital health technologies. Accumulating evidence (17, 18) suggests that digitally supported self-management interventions are associated with improved BP control and may contribute to favorable clinical outcomes, including reduced cardiovascular risk and hospitalization rates.

Overall, hypertension self-management is a comprehensive process that integrates dietary regulation, physical activity, medication adherence, and behavioral modification. However, traditional management models often lack individualized and continuous support, making it difficult to sustain long-term behavioral changes across different patient populations. These limitations may contribute to suboptimal adherence and less effective BP control, highlighting the need for more adaptive and technology-enabled approaches to support self-management.

## 4 Digital health technologies and applications in hypertension self-management

Digital health interventions for hypertension self-management involve a range of technologies designed to support continuous monitoring, behavioral intervention, personalized management, and remote healthcare delivery. These technologies integrate monitoring systems, intelligent analytical approaches, and interactive intervention strategies to facilitate patient-centered and continuous hypertension management. Current applications mainly focus on blood pressure monitoring, lifestyle modification, medication adherence, and personalized behavioral support.

### 4.1 Wearable devices and mobile health platforms

#### 4.1.1 Wearable devices

Wearable devices represent an important component of digital hypertension management. With advances in sensing technology and ambulatory cardiovascular monitoring, these devices enable the continuous acquisition of physiological parameters outside clinical settings, particularly BP-related signals. Compared with conventional intermittent measurements, wearable technologies may provide more dynamic and longitudinal data to support hypertension management. These features suggest potential benefits in early risk detection, stratified management, and treatment response monitoring. However, the clinical utility of wearable devices may be influenced by factors such as device accuracy, user adherence, data reliability, and variability in monitoring protocols.

Recent work by Yen and Huang (19) suggests that wearable technologies may support hypertension self-management by enhancing BP monitoring and facilitating behavioral change. Their pilot study using commercial smartwatches with BP-monitoring functions reported modest improvements in BP levels and self-management behaviors. In addition, evidence from meta-analyses (20) indicates that wearable-based monitoring systems, particularly when combined with remote feedback or clinical support, may contribute to small reductions in BP. Nevertheless, the reported antihypertensive effects remain relatively modest and heterogeneous across studies. Moreover, many existing studies are limited by small sample sizes, short intervention durations, and differences in device accuracy and study design. These findings suggest that wearable technologies may be more effective when incorporated into comprehensive digital health strategies rather than used as standalone interventions.

Despite promising feasibility, several challenges remain. In particular, measurement stability, calibration standardization, and optimal recalibration intervals have not yet been fully established, limiting widespread clinical adoption. In the future, further large-scale and long-term studies are needed to better establish their effectiveness and applicability in routine clinical practice.

#### 4.1.2 Mobile health applications and platforms

Mobile health (mHealth) refers to the use of mobile devices, such as smartphones, tablets, and wearable systems, to deliver health-related services and information (21). These platforms enable functions such as remote monitoring, appointment scheduling, online consultation, medication reminders, and real-time BP feedback, thereby extending care beyond traditional clinical settings.

Evidence from community-based studies indicates (22) that digital health platforms integrating data monitoring, individualized feedback, and continuous interaction may partially compensate for limitations in traditional hypertension management, particularly regarding continuity and personalization. Furthermore, mHealth-based interventions have demonstrated beneficial effects in supporting dietary management, lifestyle modification, physical activity adherence, and medication management among patients with hypertension. Evidence from Liu et al. (23) indicates that mHealth-based interventions may improve dietary behaviors in patients with hypertension, particularly adherence to low-sodium diets, while also supporting medication adherence and broader lifestyle modification.

Studies suggest that mobile applications based on the Dietary Approaches to Stop Hypertension (DASH) diet may improve adherence to healthy dietary patterns and contribute to BP reduction (24). In addition, evidence from recent studies (25–27) indicates that mobile and web-based lifestyle interventions may improve BP control, weight management, psychological well-being, and quality of life through behavior reinforcement and continuous self-monitoring.

Digital behavioral interventions delivered through social media and mobile platforms have also shown potential for improving physical activity adherence and self-management behaviors among patients with hypertension (28–30). Similarly, mobile health interventions incorporating medication reminders, adherence tracking, and clinician feedback may contribute to improved medication adherence and more favorable BP control outcomes compared with usual care (31–33).

Overall, current evidence indicates that mHealth platforms provide scalable and flexible approaches for chronic disease management, particularly in primary care and community-based settings. However, substantial heterogeneity in intervention design, duration, and population characteristics continues to limit the generalizability of findings.

### 4.2 Artificial intelligence–assisted management

Artificial intelligence (AI) leverages computational power, advanced algorithms, and large-scale datasets to support clinical decision-making and predictive modeling (34). In hypertension management, AI-based approaches have been increasingly applied to analyze patient behavior, predict medication adherence, identify risk factors associated with poor disease control, and support personalized intervention strategies.

Machine learning algorithms, including random forest, artificial neural networks, logistic regression, and XGBoost, have demonstrated superior performance in identifying non-adherent patients and capturing complex psychological and behavioral determinants compared with traditional statistical methods (35, 36). Emerging AI-enabled digital health platforms integrating lifestyle guidance, BP monitoring, and behavioral analytics have further demonstrated potential for achieving sustained improvements in BP control and long-term self-management.

These findings suggest that AI technologies may facilitate the development of data-driven personalized interventions and optimize self-management strategies for patients with hypertension. Nevertheless, challenges related to algorithm interpretability, data quality, model generalizability, and ethical considerations remain important barriers to large-scale clinical implementation.

### 4.3 Virtual reality and emerging technologies

Virtual reality (VR) is defined as an interactive, computer-generated simulation environment that allows users to engage in immersive experiences resembling real-world settings. In chronic disease management, VR has been explored as an innovative tool for patient education, behavioral intervention, and exercise promotion (37).

Recent work by Jiravská Godula et al. (38) suggests that VR-based educational interventions can improve patients’understanding of hypertension compared with conventional educational approaches. The immersive nature of VR appears to enhance disease perception and facilitate more effective knowledge acquisition.

In addition, emerging evidence (39) suggests that VR-based exercise interventions can effectively promote moderate-intensity physical activity among older adults while demonstrating favorable safety and feasibility profiles. By improving engagement and exercise adherence, these interventions may indirectly contribute to improved BP control.

Although the application of VR in hypertension management remains at an early stage, its potential in exercise-based behavioral intervention is particularly promising. VR-enabled activities, including guided aerobic exercise, interactive exergaming, balance training, and low-impact resistance exercises, can be tailored to individual fitness levels and clinical conditions, thereby supporting sustained physical activity and long-term behavioral modification.

### 4.4 Current challenges and future perspectives

Overall, digital health technologies integrate wearable devices, mobile health platforms, artificial intelligence, and virtual reality into a patient-centered and continuous management model. This paradigm shifts hypertension management from episodic monitoring toward individualized, adaptive, and technology-assisted intervention, with combined effects on behavioral modification, treatment adherence, and self-management support.

However, current evidence remains largely derived from small-scale or short-term studies, with limited long-term validation. Key barriers to clinical translation include system integration challenges, limited AI interpretability, device accessibility constraints, digital literacy disparities, and concerns regarding data privacy and security.

Future research should focus on large-scale, multicenter, and longitudinal studies to evaluate long-term effectiveness and sustainability. In addition, improvements in multimodal integration, human–machine interaction, model explainability, and personalized adaptive intervention strategies may further enhance usability, patient engagement, and clinical applicability in hypertension self-management.

## 5 Challenges and countermeasures of digital health interventions in hypertension self-management

### 5.1 Challenges and strategies in special populations

Although digital health interventions offer important advantages for hypertension management, their application in special populations remains challenged by several practical barriers. Older adults represent a particularly vulnerable group. Reduced cognitive function, limited digital literacy, and low acceptance of new technologies often prevent independent use of digital health tools (40, 41). In some cases, the absence of family support further limits patients’ ability to operate mobile health applications or wearable devices effectively. In addition, sensory impairments such as visual or hearing decline may further increase the difficulty of technology use. Beyond the older adults population, individuals in rural or resource-limited settings face additional structural barriers. Inadequate digital infrastructure, limited access to smart devices, and insufficient internet coverage may restrict the implementation of digital health interventions. Moreover, differences in cultural background and health literacy may influence the understanding and uptake of digital health content, potentially reducing intervention effectiveness.

Addressing these challenges requires multi-level and coordinated strategies rather than isolated technical improvements. From a design perspective, user-centered optimization is essential. Simplified interfaces, voice-assisted interaction, and high-contrast, large-font displays may improve usability among older adults. From a service delivery perspective, involving family members or caregivers in the intervention process may help compensate for patients’ limited operational capacity. In resource-constrained settings, low-technology alternatives such as SMS-based reminders and telephone follow-ups may provide feasible and scalable solutions. Furthermore, stratified intervention design based on age, education level, and digital literacy may improve the precision and acceptability of digital health programs.

Overall, bridging the digital divide in hypertension self-management requires the integration of technological innovation with health equity considerations. By improving usability, strengthening social support systems, and enhancing primary healthcare infrastructure, digital health interventions may become more accessible and effective across diverse populations.

### 5.2 Information security risks in digital health interventions

Despite the rapid expansion of digital health technologies in hypertension management, information security and data privacy have emerged as important concerns that may limit their large-scale implementation. Digital health interventions rely heavily on the continuous collection, transmission, and storage of sensitive health-related data, including BP measurements, medication records, and behavioral patterns (42). This extensive data exchange increases the potential risk of unauthorized access, data leakage, and cyberattacks. In particular, wearable devices and mobile health applications often depend on cloud-based platforms, which may further expose patient data to security vulnerabilities if adequate protection mechanisms are not in place. In addition, the integration of artificial intelligence and big data analytics in hypertension management raises further concerns regarding data governance and algorithm transparency. Although regulatory challenges remain, emerging legal frameworks such as the European Union Artificial Intelligence Act (EU AI Act) have begun to establish standardized requirements for AI governance, data protection, transparency, and risk management in digital health technologies. These regulations are expected to guide the development of national implementation policies in the coming years. Nevertheless, differences in regional implementation and platform-specific standards may still result in inconsistent levels of data protection across digital health systems. Moreover, patients may have limited awareness of how their personal health data are collected, stored, and utilized, which can affect trust and willingness to engage with digital health systems. From a clinical implementation perspective, insufficient interoperability between digital platforms and electronic health record systems may also increase the risk of fragmented data management and inconsistent information flow, further complicating secure data integration.

To address these challenges, strengthened data protection regulations and standardized security protocols are required. Encryption technologies, secure authentication mechanisms, and privacy-preserving data processing methods should be integrated into digital health systems. In addition, establishing clear governance frameworks for data ownership, access control, and ethical use is essential to ensure patient trust and system reliability.

Overall, while digital health technologies offer substantial benefits for hypertension self-management, ensuring information security and protecting patient privacy remain critical prerequisites for their sustainable and large-scale adoption.

### 5.3 Weaknesses in coordination mechanisms of digital health interventions

Although digital health interventions for hypertension management are rapidly evolving, the overall coordination mechanism remains underdeveloped, which to some extent limits the continuity and effectiveness of long-term disease management. At present, most digital health interventions are implemented within isolated platforms or single institutions. Cross-level collaboration between tertiary hospitals and primary care facilities is still insufficient, leading to fragmented care pathways and incomplete information transfer during patient transitions. This lack of integration weakens continuity in hypertension management and may compromise long-term clinical outcomes. In addition, heterogeneity in data structures, interoperability standards, and application programming interfaces (APIs) across different digital health platforms further restricts effective data integration. As a result, patient-generated health data are often fragmented and underutilized, limiting their potential to support clinical decision-making and population-level health management. Another important issue is the unclear role definition of healthcare professionals in digital health–supported interventions. Frontline clinicians and nurses often face increased workload due to the additional time and effort required to operate digital tools and interpret patient-generated data. However, corresponding adjustments in responsibility allocation, workflow redesign, and incentive mechanisms remain insufficient, which may negatively affect implementation efficiency and sustainability.

Taken together, these challenges highlight that the current digital health coordination system still requires substantial improvements in organizational structure, data standardization, and multi-stakeholder engagement. From a systems perspective, strengthening interoperability between information systems, standardizing data exchange protocols, and redesigning clinical workflows are essential steps for improving integration efficiency. More importantly, fostering a stable and clearly defined collaborative network among hospitals, primary care institutions, healthcare professionals, patients, and caregivers is crucial. Only when a coordinated, multi-level, and sustainable care network is established can the full potential of digital health technologies in hypertension management be realized in real-world clinical practice (see Table 1).

**Table 1:** Summary of randomized controlled trials on digital health interventions for hypertension self- management.

Category	Author	Study design	Digital intervention	Sample size	Duration	Key outcomes
Wearable & monitoring technologies	Yen and Huang (19)	RCT	Commercial smartwatch with BP monitoring feature	*n* = 60	12 weeks	↓ SBP and ↓ DBP in intervention group; significant group- by-time effect for DBP
VR intervention	Jiravská Godula et al. (38)	RCT	Virtual reality-based hypertension education	*n* = 60	4 weeks	Improved hyper- tension knowledge and patient engagement
mHealth app	Liu et al. (23)	RCT	Mobile health app for self- management	*n* = 297	6 months	Significant BP reduction and improved. self-management behavior
mHealth app	Beger et al. (26)	RCT	App-based lifestyle + BP management program	*n* = 139	12 weeks	Improved BP control and lifestyle adherence
mHealth app	Lee et al. (27)	RCT	Mobile app self management + psychological support	*n* = 100	6 months	Reduced BP and improved mental health outcomes
WeChat-based intervention	Li et al. (28)	RCT	WeChat-based behavioral intervention	*n* = 68	12 weeks	Improved health behaviors and BP control
Digital health platform	Liu K et al. (29)	RCT	Fitness app + gamification	*n* = 547	12 months	Significant reduction in SBP, improved physical activity adherence and lifestyle behaviors; modest but clinically meaningful BP improvement
mHealth + wearable	Zhang et al. (30)	RCT	Smartphone app + wearable BP monitoring	*n* = 134	12 weeks	Significant BP reduction in rural hypertensive patients
mHealth app	Arshed et al. (31)	RCT	Multifaceted mHealth package (medication + reminders + education)	*n* = 439	6 months	improved medication adherence and reduced BP
mHealth app	Morawski et al. (32)	RCT	Smartphone-based medication adherence application	*n* = 412	12 weeks	Improved antihypertensive medication adherence;

BP, blood pressure; SBP, systolic blood pressure; DBP, diastolic blood pressure.

## 6 Conclusion

Digital health interventions leveraging mobile health, wearable devices, artificial intelligence, and related technologies may support hypertension self-management through continuous monitoring, individualized intervention delivery, and improved adherence to healthy behaviors. Future development should prioritize patient-centered and stratified digital health systems, incorporating adaptive feedback and condition-specific reminder mechanisms tailored to blood pressure levels, comorbidities, and medication regimens. Particular attention should be given to usability and accessibility for older adults and populations with limited digital literacy.

Despite the growing evidence base, the translation of digital health interventions into routine clinical practice remains challenging. Real-world implementation is influenced by multiple factors, including patient digital literacy, long-term engagement and adherence, healthcare provider acceptance, and concerns regarding data privacy and security. In addition, disparities in digital infrastructure and healthcare resources may limit equitable access across different populations. Interoperability with existing electronic health record systems and reimbursement mechanisms also plays a critical role in determining scalability and sustainability.

Therefore, while digital health technologies hold considerable promise for improving hypertension management, their real-world effectiveness depends not only on clinical efficacy but also on system-level readiness and contextual factors that influence large-scale implementation.
